# Mastoiditis: Indications for Operation and Report of Some Interesting Cases

**Published:** 1912-03

**Authors:** C. W. Canan

**Affiliations:** Orkney Springs, Va.


### MASTOIDITIS; INDICATIONS FOR OPERATION AND REPORT OF SOME INTERESTING CASES.

By C. W. Canan, B. S., M. D., Ph. D., Orkney Springs, Va.

This article will be devoted to the symptomatology of the disease with the clinical report of recent cases with remarks.

*Discharge* from the middle ear is invariably present, either for some time before or immediately preceding the mastoid involvement. This discharge may be continuous or intermittent in character. *Pain* is usually present, but in a few cases may be so mild as to be overlooked. It is generally spontaneous and severe in character. *Tenderness,* either of the whole or some portion of the mastoid, is generally present, greatest at the apex. *Perforation* of the tympanic membrane is seen in about one-half the cases. Its position generally is posterio-superior in position. In about one-third of the cases, the perforation has a pouting or teat-like appearance.

*Tinnitus* occurs in at least fifty per cent. of the cases. In the writer's experience the per cent. of head noises are very much higher than this. They are of every kind, but the majority of patients claim them to be pulsating in character and describe them as "beating," "puffing," "blowing, etc., etc. *Swelling.*—In about one-fourth of these cases, the posterior superior wall of the external auditory anal will be found bulging into the canal. So great is this in a few cases that the view of the tympanum is entirely obliterated.

Swelling over the mastoid is also seen in many cases. It may be so little as to be scarcely recognized, or so great as to push the auricle outward and foreward.

Mastoiditis is no respector of age. It has been seen in infants only a few months old and in men of seventy years. It seldom if ever occurs as a disease *per se,* but comes as a complication or sequela of some former disease: La Grippe, otitis media, the exauthema and tuberculosis are generally responsible. Anything that reduces the vital forces predisposes to the disease.

There should be some way or means of educating mothers and nurses that to neglect "running of the ears" in babies and children, "otitis media," is to subject them to this grave and often fatal condition. It is remarkable how nature (in some of these neglected cases), has saved the subject from mastoiditis. A few years ago I treated a young man with chronic middle ear trouble, that dated back to his babyhood. He said that his parents had asked advice about the child's running ears, but were told that he would outgrow it. All these years the trouble had gone on until his hearing was badly impaired for life. The discharge was so offensive that flies followed him during hot weather. There was only a ragged fringe to indicate where the tympanic membrane had been. Caries had affected the bones in one ear and they were partially gone. In the other they were ankylosed and the midde ear was filled with granulations that bled on the slightest touch. We could fill a page with the damage done and yet nature had saved him from mastoiditis.

In another case the mother asked the writer to give her something for the baby. That one ear had been running for a few days and smelled badly; that the baby was very restless and cried a great deal. On the following day I was called, found the child delirious; the external auditory canal was so swollen that it was impossible to get a view of the drum; the ear was swollen stiff and pushed forward. Over the mastoid the swelling was great for that region and very sensitive. The skin was a deep purple in color over the apex. Mastoiditis was diagnosed and an immediate operation advised. The parents were ignorant and would not consent to an operation. The region was depleted by local measures, and the best suppository treatment used internally, but to no avail. The little patient went into a profound coma and finally died on the third day in convulsions, just one week from the time there was noticed any trouble with the ear.

In treating mastoiditis, a fair rule to follow will be found in using abortive measures for from two to six days; this will cure a fair proportion of cases, if seen early. *If the symptoms do not abate, but continue to increase in severity, do not persist in abortive meatures, but operate.* Especially is this true of the external manifestations, pain and tenderness, redness and odema over the mastoid, the bulging postero-superiorly into the external canal and the pushing outward of the auricle.

It should be remembered that while waiting for these symptoms to become prominent, we give the pus the same opportunity to break through the internal, as well as the external mastoid cortex; the patient's life therefore being jeopardized to the same extent.

While the operation has been considered a grave one, this is not the case, if performed with due care by skilled hands; in fact, it is relatively free from danger. *Danger to the patient arises, not from the operation, but from delay in performing it.*

The following case we report, believing that it will be of interest to the profession because of the way it terminated. Mr. M. B., farmer, 65 years old, a descendant of stout German stock, was stricken with La Grippe about the middle of December. One relapse after another confined him mostly to the house until March. He then began to look after his farm work. At the end of a week he found himself suffering from a severe ear-ache. This the writer could only relieve temporarily. During the third night the drum perforated and the ear discharged. This relieved the pain; the usual treatment was used and everything seemed to be progressing favorably until the fifth day, when pain set in again, this time more intense than before. Within three days many of the symptoms of mastoiditis were present. We sent him to a surgeon in Harrisonburg, the diagnosis was confirmed and an immediate operation was advised. The patient was afraid of the anaesthetic and came back as he went. One week passed, the writer doing all he knew, and the patient suffering terribly except when under some powerful opiate. We sent him back to Harrisonburg. The surgeon told him that a radical operation was the only chance and even with this the diagnosis was grave. He returned a second time as he went. The external canal was nearly swollen shut, the ear was red and pushed forward, and the tissue over the mastoid was dark red and boggy. There was a strong pulsation in the post-auricular artery; in fact, a throbbing. There was also something (if not the artery), but in close proximity to it, that under the finger gave one the impression of a small rubber tube filled with fluid under pressure. It appeared to be near the size of one's little finger and run down the neck some distance. By exerting some force over it with the fingers it seemed to dent inward but would spring out quickly as pressure was removed. The throbbing was so strong that we were undecided whether this was an overcrowded vein and receiving the pulsation from the artery or some other condition. Except this no fluctuation could be detected. The patient for days had been begging us to lance and see what we could find.

We contended that it was not good surgery. The patient was desperate with pain. Said before witnesses that he would take all responsibility. Making the site aseptic and with a sterilized lance we set it over the springy object and to one side as we thought of the artery. Click went the lance, at the same time the blood gushed out over our hand and down over patient's shoulder. To our gratification we saw it was venous, and we let it flow, demonstrating to ourselves from time to time that we could control it by pressure. While we had no way of measuring, but we believe at least a quart was extracted. By the time we had applied pressure and dressed it antiseptically, the patient said he was free from pain. The writer saw him the following morning and such a change in the parts; the swelling and redness had gone; the auricle was wrinkled and had settled back to its accustomed place. Patient had had a good night an was free from pain. The only thing abnormal was a hard roll running down the neck two and one-half inches, which was a little tender to the touch. The pulsation or throbbing was all gone. The thickened roll running down the neck disappeared in about a week. The patient had no further trouble. Went to work in about one week. Nearly one year has now passed by and still no indication of further trouble.

We want the Editor to give us his opinion of this case.

Did we have a genuine mastoiditis? If so, would a severe bleeding cure other cases? Was it the post-auricular vein we opened? Had we opened the artery would we have had to tie it to control the hemorrhage?

Was the roll left after lancing running down the neck a clot in the vein?

Jany. 25, 1912.

